# Bariatric surgery and health outcomes: An umbrella analysis

**DOI:** 10.3389/fendo.2022.1016613

**Published:** 2022-10-28

**Authors:** Jing Liao, Yiqiong Yin, Jing Zhong, Yanjun Chen, Yanbing Chen, Yue Wen, Zhaolun Cai

**Affiliations:** ^1^ Division of Gastrointestinal Surgery, Department of General Surgery, West China Hospital of Sichuan University, Chengdu, China; ^2^ West China School of Nursing, Sichuan University, Chengdu, China; ^3^ Gastric Cancer Center, West China Hospital of Sichuan University, Chengdu, China

**Keywords:** bariatric surgery, health outcomes, metabolic surgery, obesity, umbrella review

## Abstract

**Background:**

There is a relative lack of data that systematically investigates the breadth and validity of the association between bariatric surgery and health-related outcomes. We aimed to evaluate the quantity, validity, and credibility of evidence regarding the association between bariatric surgery and health-related outcomes using an umbrella review of meta-analyses.

**Methods:**

We systematically searched PubMed, Embase, and the Web of Science databases from inception until December 2, 2021, to identify meta-analyses of observational or interventional studies that investigated the association between bariatric surgery and multiple health outcomes. We extracted the summary effect size and 95% confidence interval (CI) data. The Assessment of Multiple Systematic Reviews (AMSTAR-2) and Grading of Recommendations, Assessment, Development, and Evaluations (GRADE) guidelines were used for methodological and evidence quality assessments, respectively.

**Results:**

Twenty-eight studies with 82 different health-related outcomes were included in this umbrella review. Beneficial effects of bariatric surgery have been observed in cancer incidence, mortality, cardiovascular risk, polycystic ovary syndrome (PCOS), anxiety symptoms, depressive symptoms, gestational diabetes mellitus, gestational hypertension, large for gestational age (LGA), macrosomia, post-term birth, risk of kidney stones, albuminuria, urinary incontinence, fecal incontinence, Barrett’s esophagus, and diabetic retinopathy. However, adverse effects of bariatric surgery were observed for maternal anemia, perinatal mortality, congenital anomalies, preterm birth, neonatal intensive care unit (NICU) admission, intrauterine growth restriction, small for gestational age (SGA), fracture risk, upper limb fracture, suicide, self-harm, and alcohol use disorder (AUD).

**Conclusions:**

Current evidence suggests that bariatric surgery improves the majority of health-related outcomes; however, caution is advised given it may increase the risk of adverse mental effects, perinatal problems, and fractures.

## Introduction

Obesity has become a global problem and its prevalence has rapidly increased in recent decades (1). Bariatric surgery has been found to be effective in promoting weight loss and obesity-related comorbidities, such as diabetes and hypertension (2). In 2018, approximately 252,000 bariatric procedures were performed in the United States, and the safety and efficacy of bariatric surgery have been confirmed through long-term clinical follow-up (3).

Despite its accepted safety, the rate of bariatric surgery remains < 1% among the eligible population in the United States (4). This low rate may be driven by questions regarding the long-term effectiveness of bariatric surgery (5). In addition, bariatric surgery may be harmful despite its benefits in terms of weight loss and diabetes remission. Compared with usual care for patients with obesity, the risk of suicide increased by 1.98 times in patients after bariatric surgery (6). A study involving 2,458 participants who were followed up for an average of 4.9 years indicated that suicidal ideation was 5.3% preoperatively and 3.8% one-year postoperatively (7). Incidences of suicide and attempted suicide occurred after an average of 3.8–3.9 years post-surgery (7). A previous meta-analysis suggested that patients had a higher risk of self-harm after bariatric surgery (8). Additionally, the incidence of AUD (9) and fracture risk (10) increased after surgery. Several meta-analyses have indicated an association between bariatric surgery and health-related outcomes; however, their results have been controversial. For example, a meta-analysis involving seven studies suggested that bariatric surgery was associated with a lower risk of cancer incidence (11). In contrast, another meta-analysis involving three studies indicated that bariatric surgery did not significantly reduce the risk of prostate and esophageal cancer (12). Furthermore, outcomes such as sexual function and PCOS have typically received less attention (13, 14).

An umbrella review is a reassessment of systematic reviews and meta-analyses on all health outcomes associated with a particular exposure (15). It provides the highest level of evidence and leads to more reliable conclusions concerning a medical research topic (16). Herein, we performed an umbrella review to identify and evaluate the association between bariatric surgery and health-related outcomes, systematically assessed the quality and strength of the evidence across all health outcomes, and identified studies with the strongest evidence.

## Methods

### Search strategy

We systematically searched PubMed, Embase, and Web of Science from inception until December 2, 2021, to identify meta-analyses of observational or interventional studies that investigated the association between bariatric surgery and any health-related outcomes. Detailed search terms are available in **Supplementary File 1**. To avoid missing relevant meta-analyses during the initial search, we manually searched the reference lists of eligible publications and applicable clinical guidelines.

### Eligibility criteria

The inclusion criteria were as follows (1): investigating the association between bariatric surgery and health-related outcomes; (2) each outcome consisting of at least three studies; (3) studies reporting effect sizes: odds ratio (OR), relative risk (RR), and hazard ratio (HR); (4) summary effect size with 95% (CI); and (5) published in English. The improvement or remission of diabetes after gastrectomy was initially reported > 50 years ago (17). A range of national and international guidelines and position statements state that bariatric surgery can lead to immediate and long-lasting diabetes remission in patients with diabetes and obesity (18–20). Owing to the well-known role of bariatric surgery in the treatment of diabetes, studies that investigated diabetes as the outcome of interest were excluded. We also excluded meeting abstracts, narrative reviews, studies with no data on health outcomes, systematic review protocols, animal studies, and other basic studies. When several meta-analyses investigated the same outcome, we selected the newest meta-analysis with the largest number of studies (21, 22). Two authors independently reviewed the studies. All differences were discussed and resolved by consensus.

### Data extraction

Two authors independently extracted all data. Disagreements were resolved by consensus. When the meta-analysis included multiple outcomes, each outcome was extracted separately. The following items were extracted from each meta-analysis: health-related outcomes, first author and year of publication, follow-up, type of bariatric surgery, number of studies and participants in the meta-analysis, design of the original studies, metric of effect size, effects model of meta-analysis, effect size with 95% CI, P-value of heterogeneity or value of *I* ^2^, and publication bias measures. If the numbers of cases and controls were not reported, we extracted these from the original study.

### Assessment of methodological quality

Methodological quality was assessed for each meta-analysis using the AMSTAR-2, a methodological quality tool used to evaluate systematic reviews and meta-analyses of randomized and non-randomized studies (23). The AMSTAR-2 consists of 16 items, seven of which are critical domains. Each review was graded on whether the critical or non-critical items had methodological defects. Grades were divided into “high”, “moderate”, “low”, and “critically low”. The AMSTAR-2 provides good agreement, reliability, construct validity, and feasibility for methodological quality assessments.

### Credibility of the evidence

The quality of health-related outcomes was assessed using the GRADE system, which offers a transparent and structured process for developing and presenting evidence summaries (24). Typically, the evidence quality of each outcome is divided into four categories (“high”, “moderate”, “low”, and “very low”) according to assessment of the risk of bias, inconsistency, indirectness, imprecision, and publication bias (25).

### Data analysis

The aim of an umbrella review is not to repeat the searches, assess study eligibility, evaluate the risk of bias, or conduct meta-analyses of the included reviews, but rather to provide an overall picture of the findings for particular questions (16). Therefore, we only extracted the existing effect size and 95% CI for each outcome rather than searching for the original studies and reanalyzing the summary estimates. The measures of heterogeneity were based on the P-value of heterogeneity or value of *I* ^2^; P-value < 0.1 or *I* ^2^ ≥ 50% were regarded as having significant heterogeneity. Publication bias was assessed using Egger’s test, Begg’s test, or funnel chart in the related meta-analysis, which indicated statistically significant publication bias when the P-value was < 0.1.

## Results

### Literature review

**Figure 1:**
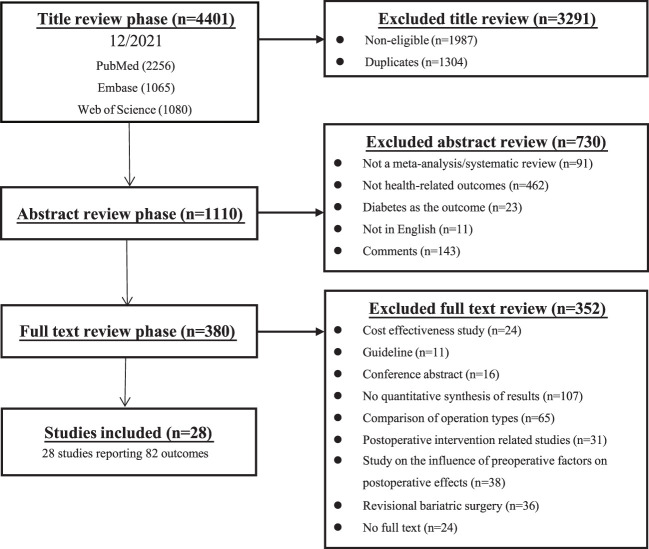
Flowchart of the article selection process.

**Figure 2:**
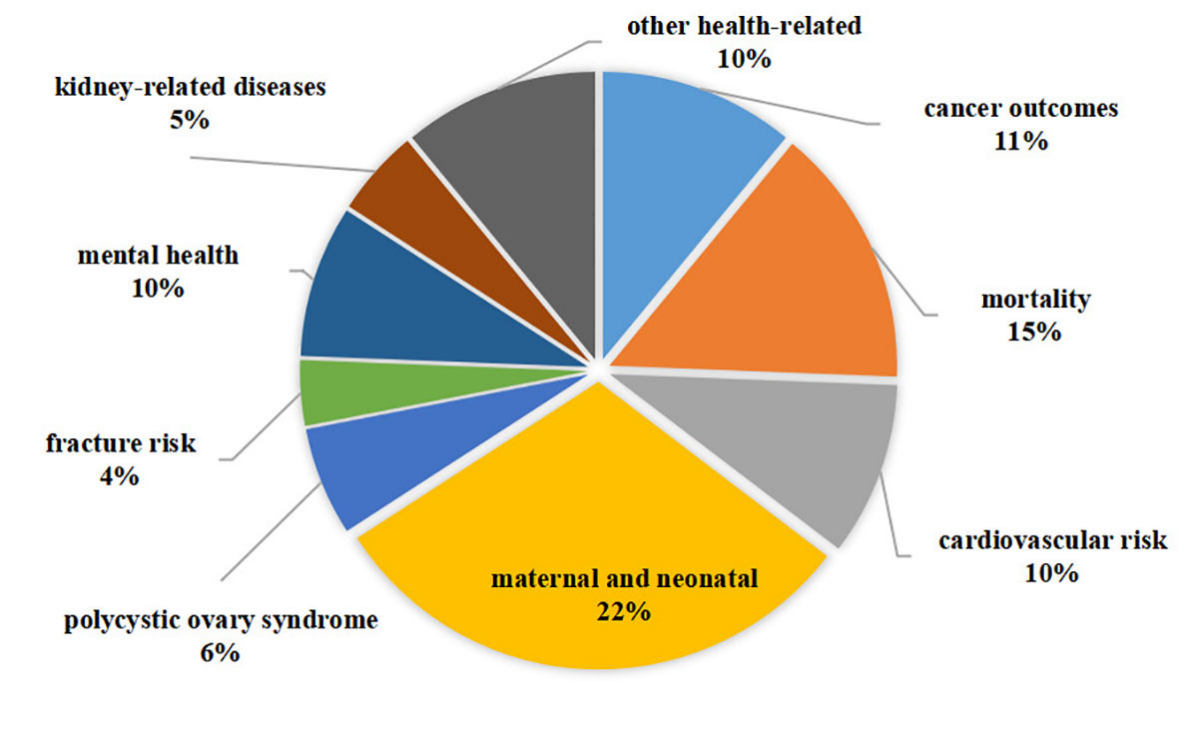
Map of outcomes associated with bariatric surgery.

A total of 4,401 potentially eligible articles were identified: 2,256 from PubMed; 1,080 from the Web of Science; and 1,065 from Embase. Eleven additional records were identified by reviewing the references of the selected studies. The flowchart of the selection process is shown in **Figure 1**. After screening titles, abstracts, and full texts, 28 studies with 82 different health-related outcomes were included in the umbrella review (**Figure 2**). The associations between bariatric surgery and health-related outcomes are presented in **Supplementary File 2**.

### Cancer risk

**Figure 3:**
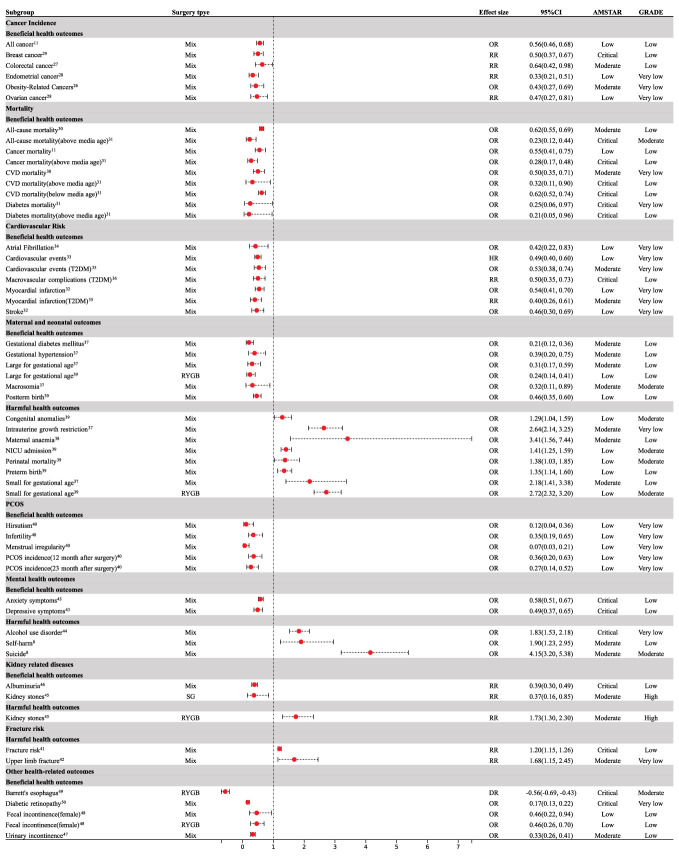
Forest plot of beneficial and harmful health outcomes associated with bariatric surgery. CI, confidence interval; CVD, cardiovascular disease; T2DM, type 2 diabetes mellitus; PCOS, polycystic ovary syndrome; RYGB, Roux-en-Y gastric bypass; SG, Sleeve Gastrectomy; NICU, neonatal intensive care unit.

**Figure 4:**
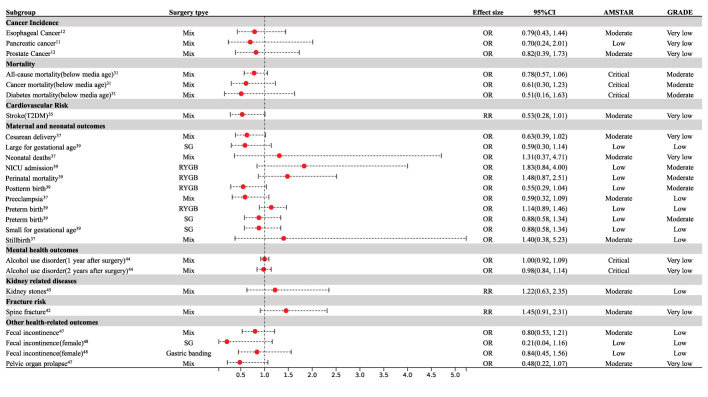
Forest plot of non-significant associations with bariatric surgery. CI, confidence interval; T2DM, type 2 diabetes mellitus; RYGB, Roux-en-Y gastric bypass; SG, Sleeve Gastrectomy; NICU, neonatal intensive care unit.

Nine meta-analyses examined the association between cancer risk and bariatric surgery; however, the results were inconclusive. Compared with patients with obesity who did not undergo bariatric surgery, patients who underwent bariatric surgery had a 44% decrease in total cancer incidence (OR = 0.56; 95% CI: 0.46 to 0.68) (11) and a reduction in obesity-related cancer (OR= 0.43, 95% CI: 0.27 to 0.69) (26) (**Figure 3**). Specifically, bariatric surgery decreased the risk of colorectal cancer (RR = 0.64; 95% CI: 0.42 to 0.98) (27), endometrial cancer (RR = 0.33; 95% CI: 0.21 to 0.51) (28), breast cancer (OR = 0.50; 95% CI: 0.37 to 0.67) (29), and ovarian cancer (OR = 0.47; 95% CI: 0.27 to 0.81) (28) (**Figure 3**). No association was found between bariatric surgery and pancreatic cancer (OR = 0.70, 95% CI: 0.24 to 2.01) (11), prostate cancer (OR = 0.82, 95% CI: 0.39 to 1.73) (12), or esophageal cancer (OR = 0.79, 95% CI: 0.43 to 1.44) (12) (**Figure 4**).

### Mortality

There was sufficient evidence that bariatric surgery significantly reduced all-cause mortality (OR = 0.62, 95% CI: 0.55 to 0.69) (30), cardiovascular disease (CVD) mortality (OR = 0.50, 95% CI: 0.35 to 0.71) (30), diabetes-related mortality (OR = 0.25, 95% CI: 0.06 to 0.97) (31), and mortality associated with cancer (OR = 0.55, 95% CI: 0.41 to 0.75) (11) (**Figure 3**). Median age at the time of the bariatric surgery was 39 years. Compared to patients with obesity who did not undergo bariatric surgery, bariatric surgery was consistently associated with a decreased risk of all-cause mortality (OR = 0.23, 95% CI: 0.12 to 0.44), CVD mortality (OR = 0.32, 95% CI: 0.11 to 0.90), diabetes-related mortality (OR = 0.21, 95% CI: 0.05 to 0.96), and cancer mortality (OR = 0.28, 95% CI: 0.17 to 0.48) above the median age (31) (**Figure 3**). Below the median age, bariatric surgery was not associated with a decrease in all-cause, diabetes, or cancer mortality (**Figure 4**), however, CVD mortality was reduced (31) (**Figure 3**).

### Cardiovascular risk

Patients who underwent bariatric surgery had a reduced risk of stroke (OR = 0.46; 95% CI: 0.30 to 0.69) (32), cardiovascular events (OR = 0.49; 95% CI: 0.40 to 0.60) (33), myocardial infarction (OR = 0.54; 95% CI: 0.41 to 0.70) (32), and atrial fibrillation (OR = 0.42; 95% CI: 0.22 to 0.83) (34) compared to those who did not undergo bariatric surgery (**Figure 3**). Bariatric surgery was also associated with a significant reduction in cardiovascular events (HR=0.53; 95% CI: 0.38 to 0.74), myocardial infarction (RR = 0.40; 95% CI: 0.26 to 0.61) (35), and macrovascular complications (RR = 0.50; 95% CI: 0.35 to 0.73) (36) in patients with type 2 diabetes mellitus over five years of follow-up (**Figure 3**). However, bariatric surgery did not reduce the incidence of stroke (RR = 0.53; 95% CI: 0.28 to 1.01) in patients with type 2 diabetes mellitus (35) (**Figure 4**).

### Maternal and neonatal outcomes

With regard to maternal outcomes, bariatric surgery reduced the rates of gestational diabetes mellitus (OR = 0.21; 95% CI: 0.12 to 0.36) and gestational hypertension (OR = 0.39; 95% CI: 0.20 to 0.75) (37) (**Figure 3**); however, bariatric surgery increased the risk of maternal anemia (OR = 3.41; 95% CI: 1.56 to 7.44) when compared with control subjects who were matched for pre-surgery body mass index (38) (**Figure 3**). Bariatric surgery was not associated with preeclampsia (OR = 0.59; 95% CI: 0.32 to 1.09) or cesarean delivery (OR = 0.63; 95% CI: 0.39 to 1.02) (37) (**Figure 4**).

Regarding neonatal outcomes, bariatric surgery was significantly related to an increased risk of perinatal mortality (OR = 1.38; 95% CI: 1.03 to 1.85), congenital anomalies (OR = 1.29; 95% CI: 1.04 to 1.59), preterm birth (OR = 1.35; 95% CI: 1.14 to 1.60), NICU admission (OR = 1.41; 95% CI: 1.25 to 1.59), intrauterine growth restriction (OR = 2.64; 95% CI: 2.14 to 3.25), and SGA (OR = 2.18; 95% CI: 1.41 to 3.38) (37, 39) (**Figure 3**). The bariatric surgery subgroup analysis demonstrated an increased risk of SGA in patients following Roux-en-Y gastric bypass (RYGB) (OR = 2.72; 95% CI: 2.32 to 3.20) (39) (**Figure 3**). Bariatric surgery significantly reduced the risk of LGA (OR = 0.31; 95% CI: 0.17 to 0.59), macrosomia (OR = 0.32; 95% CI: 0.11 to 0.89), and post-term birth (OR = 0.46; 95% CI: 0.35 to 0.60) (37, 39) (**Figure 3**). Subgroup analysis by type of surgery demonstrated that RYGB reduced LGA (OR = 0.24; 95% CI: 0.14 to 0.41) (39) (**Figure 3**). SG was not associated with SGA (OR = 0.88; 95% CI: 0.58 to 1.34) and LGA (OR = 0.59; 95% CI: 0.30 to 1.14) (39) (**Figure 4**). RYGB was not associated with perinatal mortality (OR = 1.48; 95% CI: 0.87 to 2.51), NICU admission (OR =1.83; 95% CI: 0.84 to 4.00), post-term birth (OR = 0.55; 95% CI: 0.29 to 1.04), or preterm birth (OR = 1.14; 95% CI: 0.89 to 1.46) (39) (**Figure 4**).

### Polycystic ovary syndrome

For PCOS, bariatric surgery led to a significantly lower risk of menstrual irregularity (OR = 0.07; 95% CI: 0.03 to 0.21), hirsutism (OR = 0.12; 95% CI: 0.04 to 0.36), infertility (OR = 0.35; 95% CI: 0.19 to 0.65), and the incidence of PCOS was significantly decreased at the 12-month (OR=0.36; 95% CI: 0.20 to 0.63) and 23-month (OR = 0.27; 95% CI: 0.14 to 0.52) follow-ups in comparison to pre-surgery (40) (**Figure 3**).

### Fracture risk

The meta-analysis followed up for an average of 2–4.9 years indicated that participants undergoing bariatric surgeries were associated with a higher risk of fractures (RR = 1.20; 95% CI: 1.15 to 1.26) (41), especially upper limb fractures (RR = 1.68; 95% CI: 1.15 to 2.45) (42) in comparison to patients with obesity who did not undergo bariatric surgery (**Figure 3**), however, there was no significant effect on spine fracture (RR = 1.45; 95% CI: 0.91 to 2.31) (42) (**Figure 4**).

### Mental health outcomes

Bariatric surgery improved depression (OR = 0.49; 95% CI: 0.37 to 0.65) and anxiety (OR = 0.58; 95% CI: 0.51 to 0.67) in patients with obesity after surgery (43) (**Figure 3**). Bariatric surgery appeared to increase the risk of suicide (OR = 4.15; 95% CI: 3.20 to 5.38) and self-harm (OR = 1.90; 95% CI: 1.23 to 2.95) during a follow-up of eight–ten years (8) (**Figure 3**). Bariatric surgery had no significant effect on AUD after one- and two-years follow-up (**Figure 4**); however, AUD incidence significantly increased (OR = 1.83; 95% CI: 1.53 to 2.178) after three years of follow-up (44) (**Figure 3**).

### Kidney-related diseases outcomes

Bariatric surgery was not significantly associated with the risk of kidney stones (RR =1.22; 95% CI: 0.63 to 2.35) (45) (**Figure 4**). However, the bariatric surgery subgroup analysis demonstrated an increased risk of kidney stones in patients after RYGB (RR = 1.73; 95% CI: 1.30 to 2.30) and a decreased risk of kidney stones after sleeve gastrectomy (RR = 0.37;95% CI: 0.16 to 0.85) (45) (**Figure 3**). Bariatric surgery significantly reduced albuminuria (RR = 0.39;95% CI: 0.30 to 0.49) (46) (**Figure 3**).

### Other health-related outcomes

For urinary incontinence, 13.4 months after surgery, the risk of urinary incontinence (OR = 0.33; 95% CI: 0.26 to 0.41) in women was significantly lower than that before surgery (47) (**Figure 3**). There was a significant reduction in fecal incontinence in women after bariatric surgery (OR = 0.46; 95% CI: 0.22 to 0.94) (48) (**Figure 3**). RYGB improved fecal incontinence (OR = 0.46; 95% CI: 0.26 to 0.70) (48) (**Figure 3**). Bariatric surgery significantly improved Barrett’s esophagus (RD = -0.56; 95% CI: - 0.69 to - 0.43) > 1 year after surgery (49) (**Figure 3**). Participants who underwent bariatric surgery had a significantly lower risk of diabetic retinopathy (RR = 0.17; 95% CI: 0.13 to 0.22) (50) (**Figure 3**). There was no significant effect on pelvic organ prolapse (OR = 0.48; 95% CI: 0.22 to 1.07) or fecal incontinence (OR = 0.80; 95% CI: 0.53 to 1.21) (47) (**Figure 4**). There was no effect on fecal incontinence following SG (OR = 0.21; 95% CI: 0.04 to 1.16) or gastric banding (OR = 0.84; 95% CI: 0.45 to 1.56) (48) (**Figure 4**).

### AMSTAR assessment and GRADE classification

The methodological quality of the 28 studies assessed using AMSTAR-2 is presented in **Supplementary File 3**. Nine studies (32%) were rated as critically low, eight studies (29%) as low, and eleven studies (39%) as moderate. The quality of evidence for each outcome was assessed using the GRADE system. Two outcomes were rated as high quality (2%), fifteen outcomes (18%) as moderate, thirty-five (43%) as low, and thirty (37%) as very low (**Supplementary File 4**).

## Discussion

In this umbrella review, we assessed 28 studies with 82 different health outcomes. According to the existing evidence, bariatric surgery benefits a sequence of health outcomes. Beneficial associations were found for the risk of various cancers, CVD, mortality, PCOS, urinary incontinence, and fecal incontinence. Moreover, we highlighted that bariatric surgery could be harmful in certain populations, leading to poor mental health outcomes, fractures, kidney stones, and adverse perinatal outcomes.

Bariatric surgery is associated with a lower incidence of female-specific cancers such as ovarian, breast, and endometrial cancers. Obesity contributes to approximately 6% of all cancers (51). Furthermore, it accounts for 14% and 20% of all cancer-related deaths in men and women, respectively, in the United States (51). Feigelson et al. conducted a cohort study including 17,998 bariatric surgery patients and 53,889 matched controls with ten years of follow-up, indicating that bariatric surgery was associated with a reduced risk of breast cancer in both premenopausal and postmenopausal women (52). According to a study involving 1,867 participants with obesity with a mean follow-up of 18.1 years in Sweden, bariatric surgery may reduce the incidence of female-specific cancers (53). The following mechanisms that lead to female-specific cancers may contribute to this association: hyperestrogenemia, insulin resistance, and chronic inflammation (54). Breast and endometrial cancers are highly sensitive to estrogen, and respond rapidly to changes. Adipose tissue expresses high levels of the estrogen-synthesizing enzyme aromatase; therefore, it is an important source of estrogen (55). Women with obesity are more likely to develop insulin resistance, which reduces the concentration of sex hormone-binding globulin in the body, resulting in an increase in bioavailability of estrogen (56, 57). Increased estrogen levels and bioavailability are associated with breast, ovarian, and endometrial cancers. The lower mortality after metabolic surgery may be due to improved statuses of diabetes, hypertension, and CVD (58).

In this umbrella review, bariatric surgery was found to be associated with a reduction in the prevalence of depression and anxiety. A prospective meta-analysis of 68 studies found that the postoperative prevalence of depression decreased by 8–74% (59). Psychosocial and physiological factors can lead to depression and anxiety. Increased body image satisfaction in patients with obesity after bariatric surgery results in improved self-esteem and self-worth (59), and these positive thoughts can improve symptoms of depression and anxiety. In contrast, insulin and leptin resistance affect the normal function of brain tissue, which can lead to depression (60). Bariatric surgery can improve insulin resistance and leptin secretion, thereby improving depression (61). However, this umbrella review also showed that bariatric surgery was associated with an increased risk of suicide, self-harm, and AUD. Recent studies have shown that patients with preoperative suicide-related psychiatric disorders and gastric bypass are more likely to commit suicide (62). Patients may have unrealistic expectations of life after surgery, and disappointment can lead to mental illness and suicide. By contrast, surgical trauma precipitates the remaining underlying psychiatric vulnerability in patients with a history of psychiatric disorders (63). Gastric bypass is more likely to cause nutritional deficiencies and serious complications than other types of procedures, thereby affecting patients’ quality of life and contributing to suicide (64). In addition, alcohol intake after gastric bypass surgery results in higher blood alcohol concentrations and an increased incidence of alcohol abuse, which can lead to impulsive behavior (65).

Despite the beneficial effects of bariatric surgery on pregnancy outcomes, some adverse effects persist. As many as 80% of patients undergoing bariatric surgery are women of childbearing age (66). Additionally, PCOS and fertility in women with obesity have been shown to improve after bariatric surgery. Therefore, pregnancy after bariatric surgery is becoming increasingly common. Poor perinatal outcomes included perinatal mortality, congenital anomalies, preterm birth, NICU admission, intrauterine growth restriction, and SGA. It has been hypothesized that adverse perinatal outcomes are mainly related to malnutrition. Malabsorption surgery bypasses the small intestine, the main site of vitamin and mineral absorption (67, 68). Studies have shown that 11–77% of women undergoing bariatric surgery develop anemia during pregnancy (69). The rate of folate deficiency after gastric bypass surgery is reported to be 16% (70), and 60–97% of pregnant patients post-bariatric surgery have vitamin D deficiencies (71, 72). Another study showed a median surgery-to-conception interval of 1.1 years, suggesting that many women may continue trying to lose weight when pregnant (73), leading to nutrient deficiencies. Patients willing to bear children after surgery must be fully informed about the possible benefits and risks of pregnancy, the appropriate interval between bariatric surgery and pregnancy, and effective postoperative nutritional support.

### Strengths and limitations

Our study has several strengths. First, we reviewed the association between bariatric surgery and 82 health-related outcomes. We summarized the outcomes that were significantly improved or deteriorated after bariatric surgery, which could increase understanding of the impact of bariatric surgery on multiple health-related outcomes and improve postoperative management. However, this study had some limitations. Different bariatric procedures are associated with different efficacies of weight loss and postoperative complications (74). Bariatric surgery applied in most outcomes were combined with mixed procedures. Due to the lack of relevant data, we did not analyze the impact of different bariatric procedures on health-related outcomes separately. The association between different bariatric procedures and multiple health-related outcomes should be investigated in the future. In addition, the methodological quality and the quality of evidence for each outcome of the included papers are not high. It is necessary to follow up on the health-related outcomes of bariatric surgery in real time to summarize the latest evidence.

## Conclusion

Bariatric surgery benefits most health-related outcomes and is worth promoting in patients with obesity. However, after bariatric surgery, caution should be exercised due to the increased risk of adverse mental and perinatal effects, fractures, and kidney stones. Furthermore, studies investigating ways to improve the postoperative management of patients that underwent bariatric surgery are required.

## Data availability statement

The original contributions presented in the study are included in the article/**Supplementary Material**. Further inquiries can be directed to the corresponding authors.

## Author contributions

JL was the project leader in the current study and wrote the manuscript. YJC and JZ searched the databases and screened the articles. YC and ZC extracted the data and conducted the statistical analyses. Finally, YY and YW reviewed and revised the manuscript. All authors contributed to the manuscript and approved the submitted version.

## Funding

This study was supported by the Sichuan Science & Technology Program (No. 2022YFS0167), West China Nursing Discipline Development Special Fund Project, Sichuan University (No. HXHL21002), West China Nursing Discipline Development Special Fund Project, Sichuan University (No. HXHL20044).

## Acknowledgments

The authors would like to acknowledge all authors of the original studies that were included in this meta-analysis.

## Conflict of interest

The authors declare that the research was conducted in the absence of any commercial or financial relationships that could be construed as a potential conflict of interest.

## Publisher’s note

All claims expressed in this article are solely those of the authors and do not necessarily represent those of their affiliated organizations, or those of the publisher, the editors and the reviewers. Any product that may be evaluated in this article, or claim that may be made by its manufacturer, is not guaranteed or endorsed by the publisher.
