# Supplementary figures and images for: Bariatric surgery and health outcomes: An umbrella analysis
Source: Front Endocrinol (Lausanne). 2022 Oct 28;13:1016613. doi: 10.3389/fendo.2022.1016613 (PMC9650489; doi:10.3389/fendo.2022.1016613)

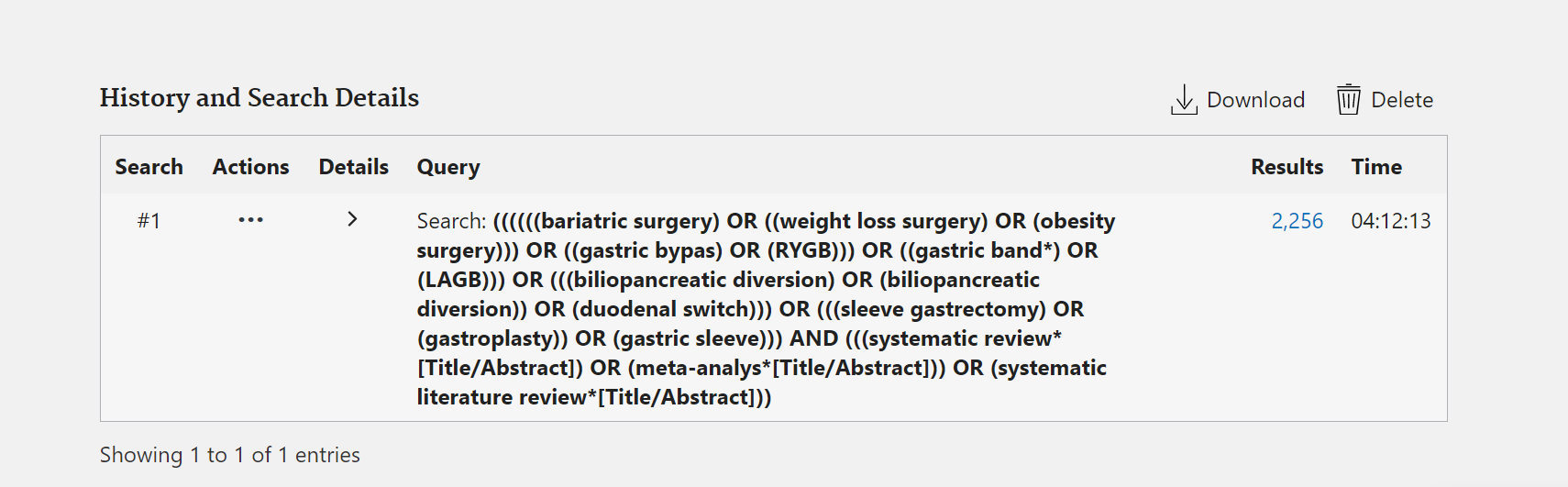

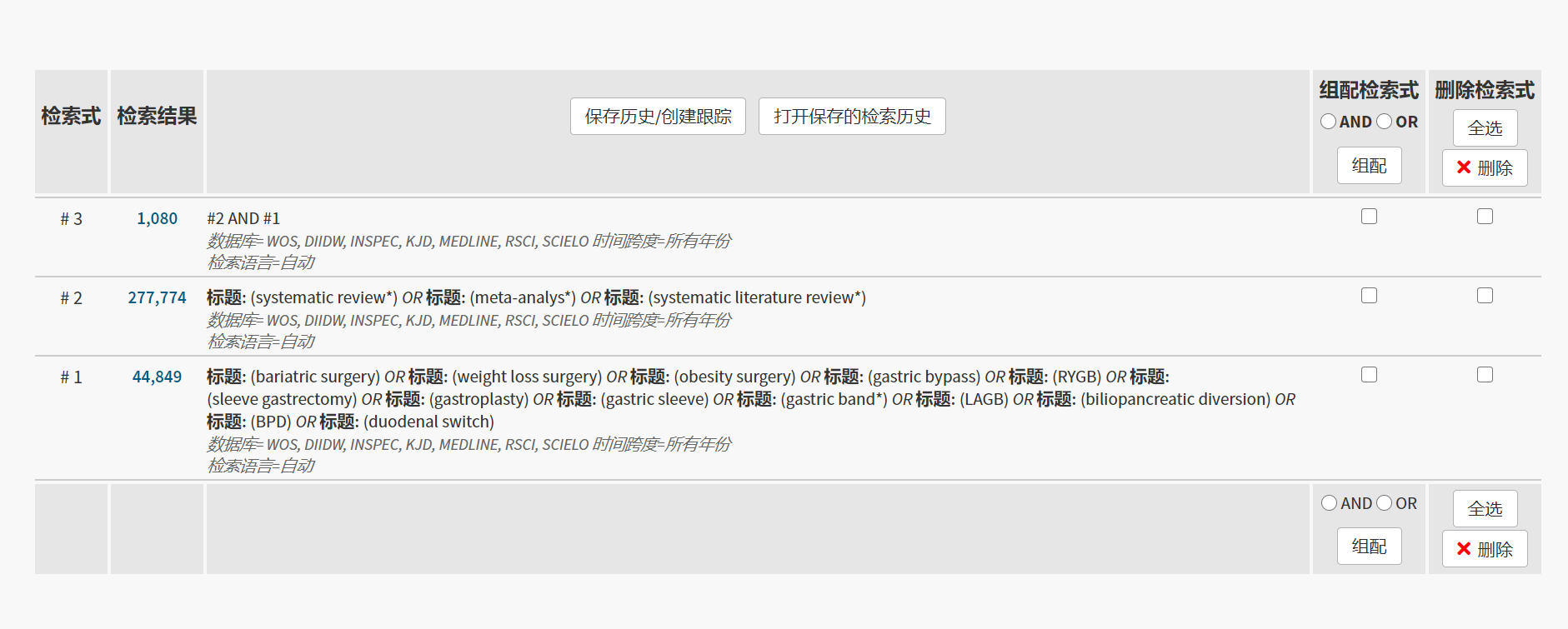

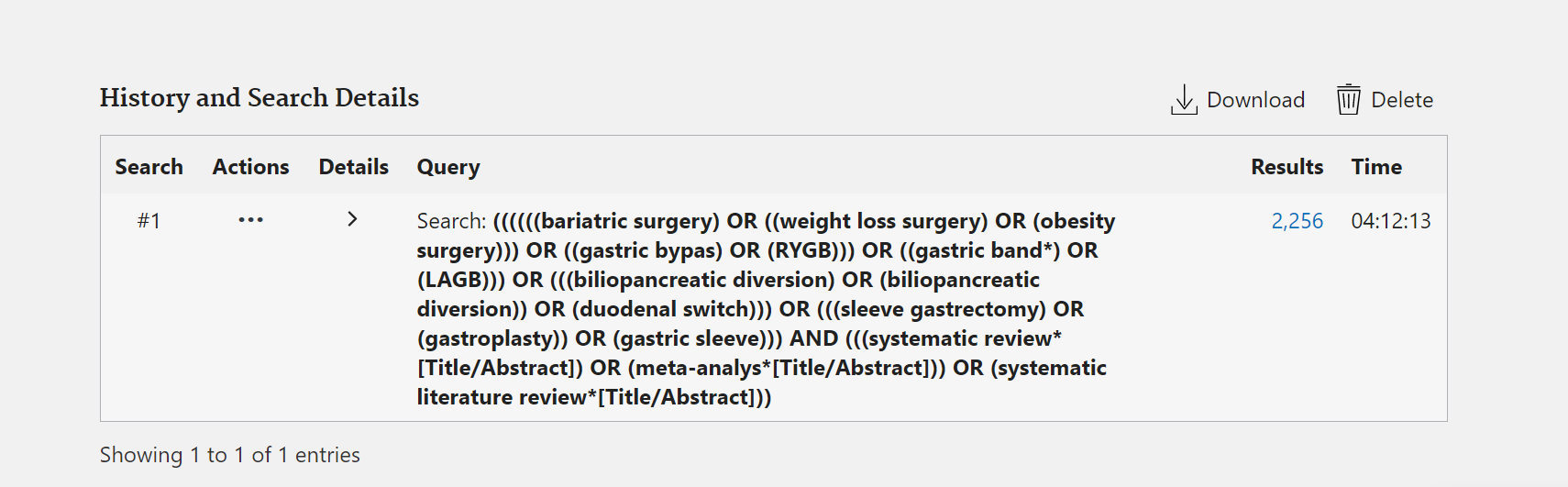

Supplement: Supplementary file 1 [file Table_1.doc]
