# Supplementary material for: Bariatric surgery and health outcomes: An umbrella analysis
Source: Front Endocrinol (Lausanne). 2022 Oct 28;13:1016613. doi: 10.3389/fendo.2022.1016613 (PMC9650489; doi:10.3389/fendo.2022.1016613)
Supplement: Supplementary file 4 [file Table_4.docx]

| **Outcome** | **First author; Year** | **Comparison** | **Type of bariatric surgery** | **Follow-up** | **Study type** | **No. of studies;**  **Participants** | **Metric**  **of MA** | **Effects**  **model** | **Effect**  **size** | **95% CI** | **I^2^ %** | **Publication**  **bias** |
| --- | --- | --- | --- | --- | --- | --- | --- | --- | --- | --- | --- | --- |
| **Cancer Incidence** | | | | | | | | | | | | |
| *Significant associations* | | | | | | | | | | | | |
| All cancer | Zhang, 2020 | Non-surgery | Mix | 3.5-25years | Cohort | 13(7981574) | OR | REM | 0.56 | 0.46 to 0.68 | 93.1% | 0.316 |
| Obesity-Related Cancers | Yang, 2015 | Non-surgery | Mix | 7-12.3years | Cohort | 5(108921) | OR | REM | 0.43 | 0.27 to 0.69 | 93% | NR |
| Colorectal cancer | Almazeedi, 2020 | Non-surgery | Mix | 3-12.5years | Cohort | 7(213727) | RR | REM | 0.64 | 0.42 to 0.98 | 85% | None |
| Endometrial cancer | Ishihara,2020 | Non-surgery | Mix | NR | Cohort | 7(1270758) | RR | REM | 0.33 | 0.21 to 0.51 | 88% | NR |
| Breast cancer | Lovrics,2021 | Non-surgery | Mix | 4.7years | Cohort | 9 (1103971) | RR | REM | 0.50 | 0.37 to 0.67 | 88% | NR |
| Ovarian cancer | Ishihara,2020 | Non-surgery | Mix | NR | Cohort | 3(5324) | RR | REM | 0.47 | 0.27 to 0.81 | 0% | NR |
| *Non-significant associations* | | | | | | | | | | | | |
| Pancreatic cancer | Zhang, 2020 | Non-surgery | Mix | NR | Cohort | 3(111444) | OR | REM | 0.70 | 0.24 to 2.01 | 62.7% | NR |
| Prostate Cancer | Wiggins,2019 | Non-surgery | Mix | NR | Cohort | 3(46749) | OR | REM | 0.82 | 0.39 to 1.73 | 77.4% | NR |
| Esophageal Cancer | Wiggins, 2019 | Non-surgery | Mix | NR | Cohort | 3(191758) | OR | REM | 0.79 | 0.43 to 1.44 | 0% | NR |
| **Mortality** | | | | | | | | | | | | |
| *Significant associations* | | | | | | | | | | | | |
| All-cause mortality | Wiggins, 2020 | Non-surgery | Mix | 59mon | Cohort | 11(898131) | OR | REM | 0.62 | 0.55 to 0.69 | 71.8% | 0.741 |
| All-cause mortality | Pontiroli,2020 | Non-surgery | Mix | 4-14years | Cohort | 9(302771) | OR ^e^ | REM | 0.23 | 0.12 to 0.44 | 97.7% | 0.127 |
| CVD mortality | Wiggins, 2020 | Non-surgery | Mix | 59mon | Cohort | 3(558960) | OR | REM | 0.50 | 0.35 to 0.71 | 29.2% | NR |
| CVD mortality | Pontiroli,2020 | Non-surgery | Mix | 5-14years | Cohort | 5(284305) | OR ^e^ | REM | 0.32 | 0.11 to 0.90 | 96.3% | 0.154 |
| CVD mortality | Pontiroli,2020 | Non-surgery | Mix | 5-14years | Cohort | 5(283623) | OR ^f^ | REM | 0.62 | 0.52 to 0.74 | 97.5% | 0.147 |
| Diabetes mortality | Pontiroli,2020 | Non-surgery | Mix | 5-14years | Cohort | 5(N) | OR | REM | 0.25 | 0.06 to 0.97 | 89.9% | 0.471 |
| Diabetes mortality | Pontiroli, 2020 | Non-surgery | Mix | 5-14years | Cohort | 5(384035) | OR ^e^ | REM | 0.21 | 0.05 to 0.96 | 89.2% | 0.460 |
| Cancer mortality | Zhang, 2020 | Non-surgery | Mix | NR | Cohort | 10(832454) | OR | REM | 0.55 | 0.41 to 0.75 | 87.3% | NR |
| Cancer mortality | Pontiroli,2020 | Non-surgery | Mix | 5-14years | Cohort | 5(284305) | OR ^e^ | REM | 0.28 | 0.17 to 0.48 | 80.3% | 0.210 |
| *Non-significant associations* | | | | | | | | | | | | |
| All-cause mortality | Pontiroli,2020 | Non-surgery | Mix | 4-14years | Cohort | 9(301787) | OR ^f^ | REM | 0.78 | 0.57 to 1.06 | 67.1% | 0.978 |
| Diabetes mortality | Pontiroli,2020 | Non-surgery | Mix | 5-14years | Cohort | 5(283623) | OR ^f^ | REM | 0.51 | 0.16 to 1.63 | 41.0% | 0.166 |
| Cancer mortality | Pontiroli,2020 | Non-surgery | Mix | 5-14years | Cohort | 5(283623) | OR ^f^ | REM | 0.61 | 0.30 to 1.23 | 48.9% | 0.380 |
| **Cardiovascular Risk** | | | | | | | | | | | | |
| *Significant associations* | | | | | | | | | | | | |
| Stroke | Kwok, 2014 | Non-surgery | Mix | NR | Cohort | 4(44988) | OR | REM | 0.46 | 0.30 to 0.69 | 79% | None |
| Cardiovascular events | Andryanto,2021 | Non-surgery | Mix | NR | Cohort | 10(1771296) | OR | REM | 0.49 | 0.40 to 0.60 | 93% | Low |
| Myocardial infarction | Kwok, 2014 | Non-surgery | Mix | NR | Cohort | 4(44988) | OR | REM | 0.54 | 0.41 to 0.70 | 58% | None |
| Atrial Fibrillation | Chokesuwattanaskul, 2020 | Non-surgery | Mix | 7.90 years | Cohort | 3(9605) | OR | NR | 0.42 | 0.22 to 0.83 | 85% | 0.28 |
| Cardiovascular events (T2DM) | Yan, 2019 | Non-surgery | Mix | ＞5 years | RCT/Cohort | 3(33106) | HR | REM | 0.53 | 0.38 to 0.74 | 0% | NA |
| Myocardial infarction  (T2DM) | Yan, 2019 | Non-surgery | Mix | ＞5 years | RCT/Cohort | 7(49302) | RR | REM | 0.40 | 0.26 to 0.61 | 62% | NA |
| macrovascular complications (T2DM) | Hussain，2021 | Non-surgery | Mix | ＞21.2mon | Cohort | 5（49211） | RR | REM | 0.50 | 0.35 to 0.73 | 71% | NR |
| *Non-significant associations* | | | | | | | | | | | | |
| Stroke(T2DM) | Yan,2019 | Non-surgery | Mix | ＞5 years | RCT/Cohort | 5(36985) | RR | REM | 0.53 | 0.28 to 1.01 | 72% | NA |
| **Maternal and neonatal outcomes** | | | | | | | | | | | | |
| *Significant associations* | | | | | | | | | | | | |
| Gestational diabetes mellitus | Kwong, 2018 | Non-surgery | Mix | NR | Cohort | 5(4034) | OR | REM | 0.21 | 0.12 to 0.36 | 49% | None |
| Gestational hypertension | Kwong, 2018 | Non-surgery | Mix | NR | Cohort | 3(462) | OR | FEM | 0.39 | 0.20 to 0.75 | 0% | None |
| Maternal anaemia | Galazis, 2014 | Non-surgery | Mix | NR | Cohort/Case–control | 4(134440) | OR | REM | 3.41 | 1.56 to 7.44 | 33% | None |
| Perinatal mortality | Akhter,2019 | Non-surgery | Mix | NR | Cohort | 10(219929) | OR | REM | 1.38 | 1.03 to 1.85 | 12.1% | 0.888 |
| Congenital anomalies | Akhter, 2019 | Non-surgery | Mix | NR | Cohort | 10(262350) | OR | REM | 1.29 | 1.04 to 1.59 | 28% | 0.218 |
| Preterm birth | Akhter, 2019 | Non-surgery | Mix | NR | Cohort | 8(1279) | OR | REM | 1.35 | 1.14 to 1.60 | 50.1% | 0.090 |
| NICU admission | Akhter,2019 | Non-surgery | Mix | NR | Cohort | 9(13035) | OR | REM | 1.41 | 1.25 to 1.59 | 0% | 0.385 |
| Intrauterine growth restriction | Kwong, 2018 | Non-surgery | Mix | NR | Cohort | 4(188057) | OR | FEM | 2.64 | 2.14 to 3.25 | NR | None |
| Small for gestational age | Kwong, 2018 | Non-surgery | Mix | NR | Cohort | 6(4991) | OR | REM | 2.18 | 1.41 to 3.38 | 50% | None |
| Small for gestational age | Akhter,2019 | Non-surgery | RYGB | NR | Cohort | 10(1291) | OR | REM | 2.72 | 2.32 to 3.20 | 0% | None |
| Large for gestational age | Kwong, 2018 | Non-surgery | Mix | NR | Cohort | 3(3924) | OR | REM | 0.31 | 0.17 to 0.59 | 77% | None |
| Large for gestational age | Akhter,2019 | Non-surgery | RYGB | NR | Cohort | 10(3164) | OR | REM | 0.24 | 0.14 to 0.41 | 70.1% | None |
| Macrosomia | Kwong, 2018 | Non-surgery | Mix | NR | Cohort | 5(4088) | OR | REM | 0.32 | 0.11 to 0.89 | 83% | None |
| Postterm birth | Akhter, 2019 | Non-surgery | Mix | NR | Cohort | 5(9259) | OR | REM | 0.46 | 0.35 to 0.60 | 7.2% | 0.148 |
| *Non-significant associations* | | | | | | | | | | | | |
| Preeclampsia | Kwong, 2018 | Non-surgery | Mix | NR | Cohort | 3(462) | OR | FEM | 0.59 | 0.32 to 1.09 | 0% | None |
| Cesarean delivery | Kwong, 2018 | Non-surgery | Mix | NR | Cohort | 4(1162) | OR | REM | 0.63 | 0.39 to 1.02 | 62% | None |
| Neonatal deaths | Kwong, 2018 | Non-surgery | Mix | NR | Cohort | 3(4442) | OR | REM | 1.31 | 0.37 to 4.71 | 59% | None |
| Stillbirth | Kwong, 2018 | Non-surgery | Mix | NR | Cohort | 4(4635) | OR | REM | 1.4 | 0.38 to 5.23 | 44% | None |
| Small for gestational age | Akhter,2019 | Non-surgery | SG | NR | Cohort | 7(1092) | OR | REM | 0.88 | 0.58 to 1.34 | 0% | None |
| Perinatal mortality | Akhter,2019 | Non-surgery | RYGB | NR | Cohort | 4(2462) | OR | REM | 1.48 | 0.87 to 2.51 | 12.1% | 0.888 |
| Preterm birth | Akhter, 2019 | Non-surgery | RYGB | NR | Cohort | 19(281729) | OR | REM | 1.14 | 0.89 to 1.46 | 9.8% | 0.090 |
| Preterm birth | Akhter, 2019 | Non-surgery | SG | NR | Cohort | 7(94) | OR | REM | 0.88 | 0.58 to 1.34 | 0% | None |
| Postterm birth | Akhter,2019 | Non-surgery | RYGB | NR | Cohort | 3(828) | OR | REM | 0.55 | 0.29 to 1.04 | 7.2% | 0.888 |
| Large for gestational age | Akhter,2019 | Non-surgery | SG | NR | Cohort | 6(1078) | OR | REM | 0.59 | 0.30 to 1.14 | 61.8% | None |
| NICU admission | Akhter,2019 | Non-surgery | RYGB | NR | Cohort | 4(680) | OR | REM | 1.83 | 0.84 to 4.00 | 0% | None |
| **PCOS(5)** | | | | | | | | | | | | |
| *Significant associations* | | | | | | | | | | | | |
| Menstrual irregularity | Skubleny, 2015 | Pre-post surgery | Mix | 23mon | Case–control | 9(3228) | OR | REM | 0.07 | 0.03 to 0.21 | 91% | NR |
| Hirsutism | Skubleny, 2015 | Pre-post surgery | Mix | 23mon | Case–control | 4(428) | OR | REM | 0.12 | 0.04 to 0.36 | 66% | NR |
| Infertility | Skubleny, 2015 | Pre-post surgery | Mix | 23mon | Case–control | 5(1122) | OR | REM | 0.35 | 0.19 to 0.65 | 0% | NR |
| PCOS incidence | Skubleny,2015 | Pre-post surgery | Mix | 12mon | Case–control | 7（4318） | OR | REM | 0.36 | 0.20 to 0.63 | 60% | NR |
| PCOS incidence | Skubleny, 2015 | Pre-post surgery | Mix | 23.mon | Case–control | 8(4705) | OR | REM | 0.27 | 0.14 to 0.52 | 78% | NR |
| **Fracture risk** | | | | | | | | | | | | |
| *Significant associations* | | | | | | | | | | | | |
| Fracture risk | de Holanda，2021 | Non-surgery | Mix | 1-17.9 years | Cohort/RCT | 10(250842) | RR | EMR | 1.20 | 1.15to 1.26 | 94% | None |
| Upper limb fracture | Zhang,2018 | Non-surgery | Mix | 2-4.9years | Case–control | 3(65559) | RR | REM | 1.68 | 1.15 to 2.45 | 76% | NR |
| *Non-significant associations* | | | | | | | | | | | | |
| Spine fracture | Zhang,2018 | Non-surgery | Mix | 2-4.9years | Case–control | 3(65559) | RR | REM | 1.45 | 0.91 to 2.31 | 41% | NR |
| **Mental health outcome(5)** | | | | | | | | | | | | |
| *Significant associations* | | | | | | | | | | | | |
| Suicide | Castaneda, 2019 | Non-surgery | Mix | 8-10 years | cohort/case-control | 5(33856) | OR | FEM | 4.15 | 3.20 to 5.38 | 15% | None |
| Self-harm | Castaneda, 2019 | Pre-post surgery | Mix | 8-10 years | cohort/case-control | 3(43,406) | OR | REM | 1.9 | 1.23 to 2.95 | 99% | None |
| Anxiety symptoms | Loh,2021 | Pre-post surgery | Mix | 3-120mon | Cohort | 26(11255) | OR | FEM | 0.58 | 0.51to 0.67 | 64.8% | None |
| Depressive symptoms | Loh,2021 | Pre-post surgery | Mix | 3-120mon | Cohort | 30(11255) | OR | REM | 0.49 | 0.37 to 0.65 | 86% | None |
| AUD | Azam, 2018 | Pre-post surgery | Mix | 3 years | cohort/case-control | 8(NR) | OR | REM | 1.83 | 1.53 to 2.178 | NR | NR |
| *Non-significant associations* | | | | | | | | | | | | |
| AUD | Azam, 2018 | Pre-post surgery | Mix | 1 year | cohort/case-control | 8(NR) | OR | REM | 1.004 | 0.92 to 1.09 | NR | NR |
| AUD | Azam, 2018 | Pre-post surgery | Mix | 2 years | cohort/case-control | 6(NR) | OR | REM | 0.981 | 0.84 to 1.14 | NR | NR |
| **Kidney related diseases (3)** | | | | | | | | | | | | |
| ***Significant associations*** | | | | | | | | | | | | |
| Kidney stones | Thongprayoon, 2016 | Non-surgery | RYGB | 3-6years | Cohortl/RCT | 3(10946) | RR | REM | 1.73 | 1.30 to 2.30 | 26% | None |
| Kidney stones | Thongprayoon,2016 | Non-surgery | SG | 2-4years | Cohortl/RCT | 3(2070) | RR | REM | 0.37 | 0.16 to 0.85 | 0% | None |
| Albuminuria | Huang,2021 | Pre-post surgery | Mix | ＞6mon | None | 23(4970) | RR | REM | 0.39 | 0.30 to 0.49 | 69% | None |
| ***Non-significant associations*** | | | | | | | | | | | | |
| Kidney stones | Thongprayoon, 2016 | Non-surgery | Mix | 2-6years | Cohort/RCT | 4(11348) | RR | REM | 1.22 | 0.63 to 2.35 | 83% | None |
| ***Other health-related outcomes*** | | | | | | | | | | | | |
| ***Significant associations*** | | | | | | | | | | | | |
| Urinary incontinence | Montenegro, 2018 | Pre-post surgery | Mix | 13.4mon | Cohort | 19(6331) | OR | REM | 0.33 | 0.26 to 0.41 | 58% | None |
| Fecal incontinence（female） | Mohamed ，2021 | Pre-post surgery | Mix | ＞23mon | Cohort | 6(607) | OR | REM | 0.46 | 0.22 to 0.94 | 75% | NR |
| Fecal incontinence（female） | Mohamed ，2021 | Pre-post surgery | RYGB | ＞23mon | Cohort | 4(302) | OR | REM | 0.46 | 0.26 to 0.70 | 32% | NR |
| Barrett’s esophagus | Adil,2019 | Pre-post surgery | RYGB | > 1 year | Cohort | 8(234) | RD | REM | -0.56 | -0.69 to -0.43 | 49% | Yes |
| Diabetic retinopathy | Caberry，2021 | Non-surgery | Mix | 1-13.2 years | Cohort/RCT | 9(334600) | RR | REM | 0.17 | 0.13 to 0.22 | 91% | Low |
| ***Non-significant associations*** | | | | | | | | | | | | |
| Pelvic organ prolapse | Montenegro,2018 | Pre-post surgery | Mix | 13.4mon | Cohort | 5(907) | OR | REM | 0.48 | 0.22 to 1.07 | 80% | NR |
| Fecal incontinence（female） | Montenegro, 2018 | Pre-post surgery | Mix | 13.4mon | Cohort | 9(1468) | OR | REM | 0.80 | 0.53 to 1.21 | 59% | None |
| Fecal incontinence（female） | Mohamed ，2021 | Pre-post surgery | SG | ＞23mon | Cohort | 4(146) | OR | REM | 0.21 | 0.04 to 1.16 | 43% | NR |
| Fecal incontinence（female） | Mohamed ，2021 | Pre-post surgery | Gastric banding | ＞23mon | Cohort | 4(146) | OR | REM | 0.84 | 0.45 to 1.56 | 0% | NR |

CI, confidence interval; CVD, cardiovascular disease; T2DM, type 2 diabetes mellitus; PCOS, polycystic ovary syndrome; RYGB, Roux-en-Y gastric bypass; SG, Sleeve Gastrectomy; NICU, neonatal intensive care unit.

^f^ below media age;  ^e^above media age;
